# Management of Regional Lymph Nodes in Clinically Node-Negative Cutaneous Squamous Cell Carcinoma of the Head and Neck: A Systematic Review & Meta-Analysis

**DOI:** 10.3390/cancers17203335

**Published:** 2025-10-16

**Authors:** Kaitlyn A. Roberts, Kaiwen Chen, Benjamin M. Wahle, Shaun A. Nguyen, Michael G. Moore, Jessica A. Yesensky

**Affiliations:** 1 Department of Otolaryngology—Head and Neck Surgery, Indiana University School of Medicine, Indianapolis, IN 46202, USA; roberkai@musc.edu (K.A.R.); bwahle@wustl.edu (B.M.W.); mmoore13@iuhealth.org (M.G.M.); jyesensky@iuhealth.org (J.A.Y.); 2 Department of Otolaryngology—Head and Neck Surgery, Medical University of South Carolina, Charleston, SC 29425, USA; chenka@musc.edu

**Keywords:** cutaneous squamous cell carcinoma, squamous cell carcinoma, head and neck, clinically node-negative, occult nodal metastasis, sentinel lymph node biopsy, elective dissection, elective nodal irradiation, skin cancer

## Abstract

Background/Objectives: Head and neck cutaneous squamous cell carcinoma (HNcSCC) has the potential to metastasize to local lymph nodes, which can significantly impact prognosis. However, the optimal management of patients with clinically node-negative (cN0) disease remains unclear. Methods: We conducted a systematic review and meta-analysis following PRISMA guidelines. PubMed, Scopus, CINAHL, and Web of Science databases were searched from inception to 7 August 2025. Two parallel searches were conducted: one to capture management strategies and outcomes of cN0 patients with HNcSCC and one to capture occult nodal metastasis rates of the same population. Results: A total of 38 studies were included. Post-excision management strategies included observation, sentinel lymph node biopsy (SLNB), elective dissection (ED), and elective nodal irradiation. The pooled rate of occult lymph node metastasis was 13.9% in 1673 HNcSCC tumors overall and 12.5% when limited to 977 high-risk tumors. Overall recurrence in the SLNB group (8.3%) was significantly lower than both the observation (16.9%, *p* < 0.0001) and ED (23.7%, *p* < 0.0001) groups. Additionally, overall mortality in the SLNB group (6.1%) was significantly lower than observation (29.9%, *p* < 0.0001) and ED (31.4%, *p* < 0.0001). Conclusions: We found that SLNB was associated with lower recurrence and mortality compared with observation and ED. While not assumed to be causative, our findings support the role of SLNB in diagnosing occult metastasis and staging disease in this population.

## Simple Summary

Cutaneous squamous cell carcinoma is a common type of skin cancer that can affect the head and neck region. This cancer has the potential to exhibit subclinical metastasis to local lymph nodes that cannot be seen or felt during a physical exam or imaging, which can worsen clinical outcomes and disease prognosis. However, choosing a management strategy for patients without obvious signs of metastasis remains unclear. We reviewed and analyzed existing research to better understand the prevalence of subclinical disease and clinical outcomes associated with different management options. Our goal is to summarize the currently available data to guide future research and improve medical decision making for patients with cutaneous squamous cell carcinoma.

## 1. Introduction

Nonmelanoma skin cancer is the most commonly diagnosed malignancy in the United States, with cutaneous squamous cell carcinoma (cSCC) accounting for approximately 20% of cases [1]. An estimated 700,000 new cases of cSCC are diagnosed annually in the United States, with global incidence continuing to rise [2,3]. The majority of cSCCs occur within the sun-exposed areas of the head and neck. Due to considerable heterogeneity in tumor behavior, ranging from indolent to highly aggressive, the risk of regional metastasis varies throughout the literature, with rates approaching 20% in high-risk tumors [4,5,6].

The National Comprehensive Cancer Network (NCCN) provides the most up-to-date, evidence-based guidelines for cSCC risk stratification, evaluation, and treatment. According to NCCN criteria, all head and neck cSCCs (HNcSCC) are considered high-risk based on anatomical location alone. Additional prognostic features are used to further stratify tumors into high-risk (diameter > 2 cm and ≤ 4 cm, depth of invasion (DOI) of 2–6 mm, presence of perineural invasion (PNI), recurrence, or patient immunosuppression) and very high-risk (diameter ≥ 4 cm, DOI > 6 mm, poorly differentiated histology, PNI involving nerves ≥0.1 mm, or evidence of lymphatic or vascular invasion) categories [5]. These features align closely with prognostic variables incorporated into the 8th edition of the *American Joint Committee on Cancer (AJCC) Cancer Staging Manual*, which emphasizes DOI and PNI as key indicators of aggressive disease [7]. These features are associated with increased risk of local recurrence and regional spread, with nodal metastasis being the strongest predictor of both recurrence and disease-specific survival [4,5,6].

Surgical excision of the primary tumor—either via Mohs micrographic surgery or standard wide local excision—remains the cornerstone of treatment. However, in the setting of clinically node-negative (cN0) disease, there is no strong consensus or high-level evidence guiding post-excision management of at-risk regional lymph nodes. Options such as observation, sentinel lymph node biopsy (SLNB), elective neck dissection, and elective irradiation are employed variably, often based on institutional protocols and physician discretion [8].

Regarding imaging, the NCCN recommends that radiographic evaluation of the cN0 nodal basin be “discussed and considered” in very-high-risk cases but does not provide firm guidance for either risk group. Elective dissection of the neck, parotid, and/or other regional nodal basins is sometimes pursued in patients with multiple high-risk features, although there is no established consensus regarding its routine application [5]. Despite a lack of randomized control trial data supporting its use in cSCC, SLNB has gained traction as a less invasive method of regional staging—largely extrapolated from its well-established role in cutaneous melanoma management [9]. The most recent version of NCCN guidelines recommends considering SLNB in cases that are recurrent or with multiple high-risk features [5]. While SLNB is generally considered a safe and feasible technique, its clinical utility in cSCC remains under investigation, and long-term outcomes data are currently limited.

Overall, the NCCN guidelines reflect the limited availability of level 1 evidence to guide the evaluation and surgical management of cN0 HNcSCC. Given the prognostic significance of nodal disease, accurate risk assessment remains critical for informing post-excision treatment planning. However, compared to mucosal squamous cell carcinomas, the epidemiology and true incidence of nodal metastasis in cSCC are poorly understood. Evidence-based strategies for nodal management are largely limited to retrospective reviews and case series. The present study aims to address this gap through a systematic review and meta-analysis of both the occult nodal metastasis rate in HNcSCC and clinical outcomes associated with different management strategies for cN0 disease.

## 2. Methods

This review was prepared and reported according to Preferred Reporting Items for Systematic Review and Meta-Analysis (PRISMA) guidelines [10]. The protocol was registered with the International Prospective Register of Systematic Reviews (PROSPERO, University of York, York, UK; protocol ID CRD420251151325).

### 2.1. Search Strategy

PubMed (U.S. National Library of Medicine, National Institutes of Health), Scopus (Elsevier), CINAHL Complete (EBSCOhost), and Web of Science (Clarivate) databases were queried from inception to 7 August 2025. The search strategies used a combination of subject headings (e.g., MeSH in PubMed) and keywords. The strategy was modified for the other databases, maintaining similar keywords and replacing MeSH search terms with subject headings. Two separate searches were conducted: one to capture management strategies and outcomes of cN0 patients with HNcSCC, and one to capture occult nodal metastasis rates of the same population. Search terms included terms relating to cutaneous squamous cell carcinoma, head and neck, sentinel lymph node biopsy, elective dissection, and occult nodal metastasis. The complete search terms and strategy are detailed in Appendix A Table A1 and Table A2.

### 2.2. Selection Criteria and Data Extraction

Inclusion criteria for the systematic review were as follows: (1) clinically node negative population, (2) cutaneous squamous cell carcinoma, (3) head and neck cases only, (4) reporting of clinical patient outcomes OR occult nodal metastasis rates. Only English-language, human, prospective and retrospective studies were included. Studies were carefully evaluated, and populations containing patients who previously underwent surgical nodal intervention were excluded.

References were exported into the review management software, Covidence (Covidence systematic review software, Veritas Health Innovation, Melbourne, Australia, https://www.covidence.org/, accessed 7 August 2025), for de-duplication and study selection. Two independent reviewers (KR and KC) individually screened studies, and a third reviewer (SAN) resolved discrepancies. Titles and abstracts were first assessed for eligibility, then full text of eligible studies was reviewed for inclusion.

Data were tabulated by two authors (KR and KC) using Microsoft Excel (version 2507). For study characteristics, we extracted the following data: first author, year of publication, study design, sample size, and interventions utilized. In addition to occult nodal metastasis rates, the primary outcomes of tumor recurrence and mortality were collected to assess the efficacy of each management strategy. Other outcomes of interest including patient demographic data, adjuvant therapies, and tumor characteristics were collected. Studies with heterogeneous populations that included other cancer pathologies (e.g., melanoma, BCC) or sites (e.g., mucosal, extremities) were only included for extraction and analysis if data were stratified by our desired population (i.e., cN0 HNcSCC).

Clinically node negative status was defined as absence of head and neck lymph nodes suspicious for nodal metastasis on clinical examination and/or radiographic imaging. Patients who underwent sentinel lymph node biopsy were also classified as cN0. It is worth noting that several studies screened included patient populations that had cN0 neck status but known to be parotid node-positive. Though these studies may have reported neck nodal occult rates and outcomes, they were ultimately decided to be cN+ populations and were excluded [11,12,13,14,15,16,17,18].

Occult nodal metastasis rates were collected from any patients with HNcSCC who were cN0 at presentation and later underwent pathologic assessment of nodal status. Occult nodal metastasis was defined as any pathologically positive lymph nodes identified after an initial determination of cN0 status through physical exam ± diagnostic imaging. Tumors were designated as high-risk if the study explicitly described the population as high-risk, or if study inclusion criteria required at least two of the following features: tumor diameter ≥ 2 cm; tumor depth of invasion ≥ 6 mm; recurrent tumor; perineural invasion; lymphovascular invasion; AJCC stage T3 or greater; or patient immunosuppression. If tumor characterization did not meet these criteria or risk was not explicitly assigned by the study, tumors were categorized as unspecified risk.

Local recurrence was defined as recurrent disease at the primary skin site, while regional recurrence included disease identified in the regional lymph nodes or extra-nodal spread. Studies that did not stratify recurrence by location were categorized as overall recurrence, and studies that did stratify were pooled to calculate an overall recurrence rate.

During meta-analysis, when studies reported medians and ranges or interquartile range, we utilized the methods described by Wan et al. and Luo et al. as recommended by the Cochrane Handbook to estimate means and standard deviations [19,20].

### 2.3. Quality Assessment

The methodological quality of each included study was assessed by two authors (KR and KC) and verified by one author (SAN). Each study was assigned a level of evidence according to the Oxford Center for Evidence-Based Medicine (OLE) criteria [21]. The Joanna Briggs Institute (JBI) critical appraisal tool was utilized for assessing risk of bias of case-series and cohort studies, comprising 10 questions for case-series and 11 questions for cohort studies answered with “yes,” “no,” or “not applicable” [22]. Risk of bias was considered acceptable if the number of “yes” responses exceeded five (>50%).

### 2.4. Data Analysis

Meta-analysis of single means (age and follow-up period) and meta-analysis of proportions (patient characteristics, tumor characteristics, metastasis rates, etc.) were performed by Comprehensive Meta-Analysis (version 4, Biostat Inc., Englewood, NJ, USA). Each measure (mean/proportion [%] and 95% confidence interval [CI]) was weighted according to the number of patients affected. The random-effect model was chosen following a heterogeneity assessment of the outcome variables. The I2 statistic was used to quantify the proportion of variability attributable to heterogeneity rather than chance, and the Cochran’s Q test (χ2) to assess the presence of statistical heterogeneity [23,24]. To evaluate the robustness of our results, we conducted a sensitivity analysis using the one-study removal method, in which each study was systematically excluded in turn to assess its influence on the overall pooled estimates. We also compared proportions between the two groups, reporting the differences (Δ%) and corresponding 95% confidence intervals. Finally, potential publication bias was assessed through visual inspection of the funnel plot and statistically tested using Egger’s test [25,26]. A *p*-value of <0.05 was considered statistically significant for all analyses.

## 3. Results

### 3.1. Literature Search

**Figure 1:**
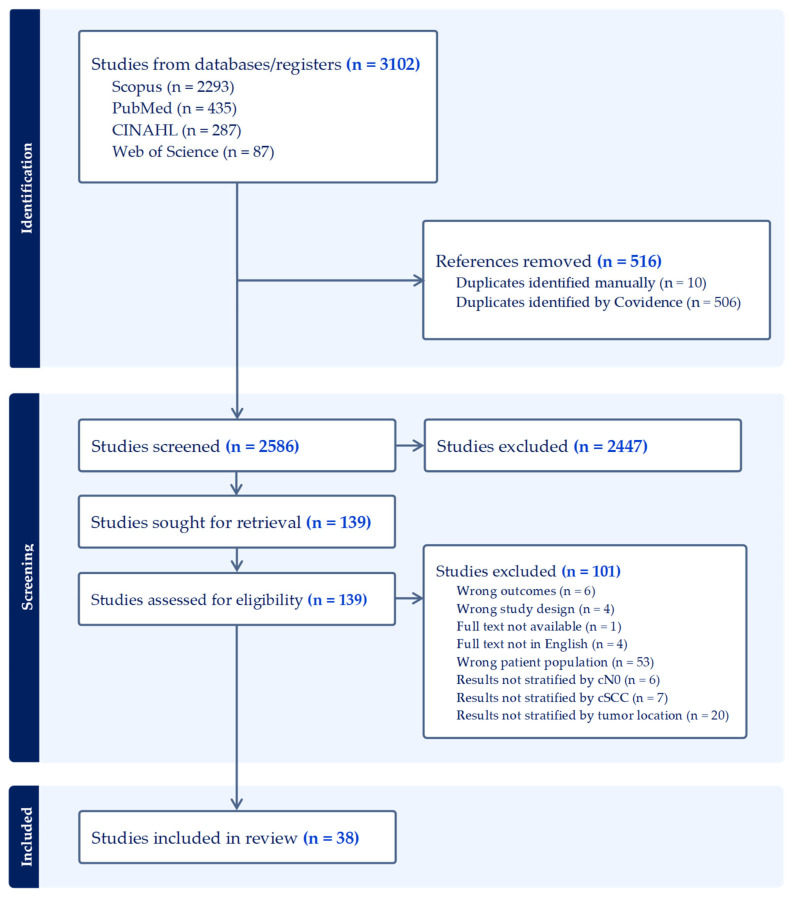
PRISMA Flow Diagram. Copyright statement: this PRISMA diagram contains public sector information licensed under the Open Government License v3.0. Adapted from: Moher et al. (2009) [27].

The initial electronic searches identified 3102 records; after removal of 516 duplicates and 2447 exclusions, 38 studies met inclusion criteria for inclusion in the systematic review and meta-analysis (PRISMA diagram, Figure 1).

The final set comprised 27 case series studies (20 retrospective, 7 prospective) and 11 cohort studies (8 retrospective, 2 prospective, 1 ambispective), between 2003 and 2025, and spanned multiple continents, including Europe, Asia, North America, and Australia [6,28,29,30,31,32,33,34,35,36,37,38,39,40,41,42,43,44,45,46,47,48,49,50,51,52,53,54,55,56,57,58,59,60,61,62,63,64].

Post-excision management strategies for cN0 HNcSCC included observation (7 studies), sentinel lymph node biopsy (SLNB, 12 studies), elective dissection (ED: including elective neck dissection and/or parotidectomy, 7 studies), and elective nodal irradiation (1 study). Because only one study analyzed elective irradiation as a management strategy, it was not included in pooled meta-analysis. Notably, 15 studies were included for their reported occult nodal metastasis rates only (Table 1).

### 3.2. Quality Assessment

Based on the OLE criteria, 11 cohort studies were classified as level 3 evidence, and 27 case series studies were classified as level 4 evidence. The 27 case series (scores 6–9) and 11 cohort studies (scores 7–11) were evaluated using the JBI Critical Appraisal Tool. All studies met inclusion criteria, indicating overall high quality (Table 2).

**Figure 2:**
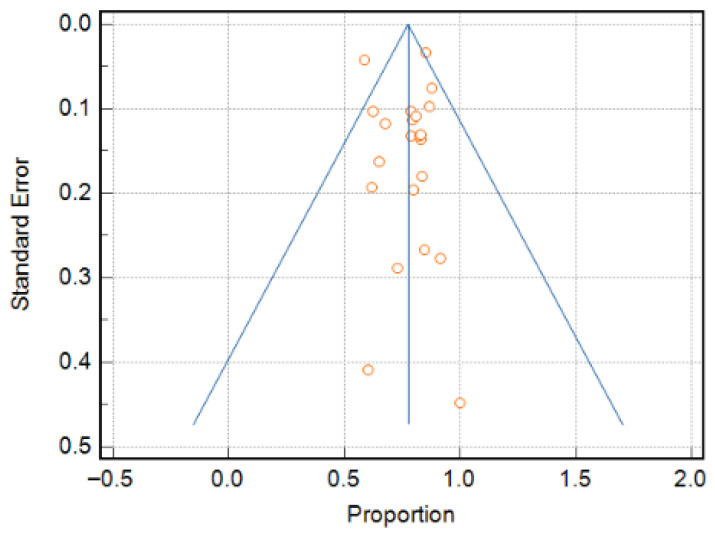
Funnel plot for assessing the presence of publication bias.

In case series studies, the most common limitation was incomplete reporting of site demographics, and many studies performed limited statistical analyses. In cohort studies, inadequate strategies to address incomplete follow-up were the most frequent limitation. Despite these shortcomings, the overall methodological quality of the included studies was acceptable, supporting confidence in the pooled findings. Finally, a funnel plot (Figure 2) with Egger’s test (−0.04, 95% CI: −2.26 to 2.18, *p* = 0.6110) suggested little publication bias, as most of the studies were within the funnel with little asymmetry [25,65].

### 3.3. Patient Demographics and Tumor Characteristics

The observation group comprised 1799 patients with 1952 tumors with a weighted mean age of 69.3 ± 0.9 years and were 75.5% male. Tumor locations were most often perioral (35.4%), unspecified (27.9%), and forehead (3.2%). AJCC stage T1-T2 tumors accounted for 73.3%, and primary tumors were more common (83.4%) than recurrent (16.6%). The most common method of adjuvant therapy was radiation (28.7%).

A total of 494 patients who underwent SLNB for 499 tumors had a weighted mean age of 68.3 ± 1.3 years and were 80.9% male. The most common tumor locations were unspecified (30.1%), perioral (25.7%), and scalp (8.7%). AJCC stage T3-T4 tumors accounted for 66.8%, and primary tumors were more common (92.5%) than recurrent (6.3%). The most common method of adjuvant therapy was radiation (21.6%).

The ED group contained 411 patients with a weighted mean age of 68.3 ± 2.1 years and were 78.0% male. Tumor locations were most often unspecified (41.7%), temple (13.3%), and ear and periauricular (9.2%). AJCC stage T1-T2 tumors accounted for 56.4% and 43.6% were AJCC stage T3-T4, with 52.9% primary and 47.1% recurrent on presentation. Adjuvant therapy consisted of radiation in 43.8% of patients and chemoradiation in 10.7%.

All pooled demographic and tumor characteristic results are reported in Table 3.

### 3.4. Occult Nodal Metastasis

Across all management strategies and tumor risk levels, analysis showed an overall occult nodal metastasis rate of 13.9% (95% CI: 10.5–17.7%) in 1673 HNcSCC tumors. Among high-risk tumors only, the rate was 12.5% (95% CI: 8.5–17.0%) in 977 tumors.

Sub-group analyses of 707 tumors managed with SLNB showed an overall occult metastasis rate of 8.8% (95% CI: 6.8–11.1%), and 8.4% in high-risk tumors (95% CI: 6.3–10.8%). For 966 tumors managed with ED, analysis yielded an overall occult metastasis rate of 17.3% (95% CI: 11.6–23.7%) and 18.8% in high-risk tumors (95% CI: 10.3–23.7%). Complete sub-group analyses of occult rates are presented in Table 4.

Direct comparison of proportions (COP) found significantly lower occult metastasis rates in the SLNB group compared with the ED group, both overall (COP: 8.5%, 95% CI: 5.3–11.6%, *p* < 0.0001) and among high-risk tumors (COP: 10.4%, 95% CI: 5.9–15.3%, *p* < 0.0001).

### 3.5. Clinical Outcomes and Comparison of Group Meta-Proportions

Overall recurrence rates were 16.9% in the observation group, 8.3% in SLNB, and 23.7% in ED. Recurrence in ED was significantly higher than observation (∆: 6.8%, *p* = 0.0084) and SLNB (∆: 15.4%, *p* < 0.0001). Recurrence in observation was also significantly higher than SLNB (∆: 8.6%, *p* = 0.0002).

Local recurrence occurred in 8.2% of observed, 11.2% of SLNB, and 21.8% of ED patients. The difference between observation and SLNB was not statistically significant (*p* = 0.3373), but local recurrence in ED was significantly greater than both observation (∆: 13.6%, *p* = 0.0103) and SLNB (∆: 10.6%, *p* = 0.0287). Regional recurrence rates for observation (6.7%), SLNB (6.8%), and ED (7.6%) were similar across management strategies. There was no significant difference between observation and SLNB (*p* = 0.9573), observation and ED (*p* = 0.5147), or SLNB and ED (*p* = 0.6444). Similarly, distant metastasis rates were similar for observation (6.7%), SLNB (4.5%), and ED (9.5%), with ED being significantly higher than SLNB (∆: 5.1%, *p* = 0.0273) but not observation (*p* = 0.3662) (Table 5).

There were no significant differences between disease-specific death for observation (5.0%), SLNB (5.6%), and ED (6.7%). Overall mortality, however, in the SLNB group (6.1%) was significantly lower than both the observation (29.9%, ∆: 23.8%, *p* < 0.0001) and ED (31.4%, ∆: 25.3%, *p* < 0.0001) groups. There was no significant difference in overall mortality between observation and ED groups (*p* = 0.6570). Complete sub-analyses of recurrence and mortality rates are presented in Table 5 and Table 6.

### 3.6. Clinical Outcomes of Elective Nodal Irradiation

Only one included study assessed the management strategy of elective nodal irradiation (ENI) and its clinical outcomes. In this retrospective case series of 71 patients with cN0 HNcSCC who underwent ENI of the regional lymph nodes, Wray et al. reported two cases of nodal recurrence with an isolated nodal recurrence rate of 2.8%. They also reported two additional cases of nodal recurrence, which were not considered ENI failures: one case recurred in a contralateral node that would not have otherwise been removed in ED, and the other recurred secondary to primary site recurrence and therefore could not be attributed to failure of ENI sterilization of subclinical disease. They also noted that nodal recurrence rate was more accurately calculated based on the number of patients who remained continuously free of primary site recurrence. After excluding those who had primary site recurrence (n = 15), they report an adjusted nodal recurrence rate of 3.6% (2 out of 56) [61].

## 4. Discussion

This systematic review and meta-analysis of 38 studies encompassing 1673 cases found an overall occult nodal metastasis rate of 13.9% and 12.5% among high-risk tumors (Table 3). SLNB showed significantly lower occult rates compared with ED and was also associated with lower recurrence and overall mortality (Table 5 and Table 6). These findings highlight SLNB as a valuable regional staging tool in management of cN0 HNcSCC.

### 4.1. Considerations for Sentinel Lymph Node Biopsy

SLNB, a fundamentally diagnostic procedure, requires multidisciplinary coordination and technical precision to ensure accurate staging. Standardization of protocols, particularly in radiotracer injections, preoperative imaging, and histopathologic evaluation, is essential. A consensus paper published in 2000 highlights best practices for SLNB technique, including serial sectioning and immunohistochemistry (IHC), both of which enhance diagnostic sensitivity [66]. In head and neck melanoma, use of SPECT/CT imaging for sentinel node localization has improved detection of nodal metastasis and supports its use in cSCC [67]. Prior studies have identified nodal metastasis in SLNB-negative specimens after re-analysis with enhanced histopathologic techniques [33]. This evidence supports the incorporation of such practices into routine SLNB protocols to avoid underdiagnosis.

Prior systematic reviews estimated SLNB positivity at 7.9% (95% CI: 5.2–10.6%) in cSCC, though these included non-head and neck tumors and did not stratify by risk level [68]. Our pooled occult nodal metastasis rate for SLNB aligns with these estimates, supporting SLNB as a minimally invasive, low-morbidity, and diagnostically reliable strategy for cN0 HNcSCC tumors. However, it must be noted that histologic techniques (e.g., serial sectioning and IHC) were inconsistently reported across the included studies, so occult rates may be underrepresented.

### 4.2. Observation Versus Sentinel Lymph Node Biopsy

Observation after tumor excision remains the most conservative management strategy and is often the favored approach for patients with lower-risk tumors to minimize the morbidity associated with more invasive procedures. However, this approach carries a risk of delayed detection of occult nodal metastasis. In our analysis, both the observation and SLNB groups primarily included patients with primary tumors. Notably, 73.3% of tumors in the observation group were staged T1–T2, while a larger proportion (66.8%) in the SLNB group were staged T3–T4, suggesting more advanced disease in the latter group.

Despite this difference, SLNB was associated with significantly lower rates of overall recurrence and mortality. Given that SLNB is a diagnostic—not therapeutic—procedure, this association should not be interpreted as evidence of a causal effect. Rather, it likely reflects the role of SLNB in early identification of occult nodal metastases, which are known to be strong predictors of prognosis in this population. By facilitating timely upstaging and appropriate treatment, SLNB may contribute to improved outcomes in appropriately selected patients. However, further prospective studies are needed to clarify these associations.

### 4.3. Considerations for Elective Dissection

Comparison between SLNB and ED is less straightforward due to significant confounding factors. The ED group had higher rates of occult nodal metastasis as well as poorer clinical outcomes, specifically local recurrence and overall mortality. However, these findings may be due to selection bias, since ED is often performed in patients with more aggressive, high-stage, or recurrent tumors. To address this limitation more clearly, we emphasize the fact that choice of management strategy in most retrospective studies was guided by surgeon preference and tumor characteristics rather than randomization. Patients who underwent elective dissection often had larger, deeper, or recurrent tumors, which inherently carry a higher risk of recurrence and mortality independent of the treatment approach. These confounding factors indicate that the observed associations may reflect underlying tumor biology and selection bias rather than a true treatment effect.

Tumor characteristics such as perineural invasion, lymphovascular invasion, or depth of invasion are also important predictors of recurrence and metastasis and may influence a surgeon’s decision to perform ED instead of less invasive options [4]. Furthermore, the influence of these features not only increases the likelihood of nodal spread, but also encourages clinicians to choose more aggressive surgical management. Therefore, the apparent benefit of SLNB and the poorer outcomes seen in the ED group should be interpreted cautiously, as these differences may reflect inherent disease aggressiveness rather than causation.

Adjuvant therapy practices, often guided by disease presentation, were also inconsistently reported among studies. The differences between the SLNB and the ED groups may therefore relate to tumor biology or adjuvant therapy selection rather than the management strategy itself. The more invasive nature of ED further complicates comparisons, highlighting the need for prospective studies and improved risk stratification.

### 4.4. Staging and Risk Stratification

Accurate staging remains critical for guiding treatment decisions and prognostication in patients with cSCC. Multiple staging systems have been proposed to stratify cSCC risk, including the American Joint Committee on Cancer (AJCC) and the Brigham and Women’s Hospital (BWH) systems. Though BWH staging has demonstrated superior prognostication for patients with localized cSCC compared to AJCC [69], current NCCN treatment guidelines continue to rely on the AJCC system as BWH does not account for regional or distant metastasis. Currently, prospective data remain limited, and there is no unified risk stratification system or standardized treatment algorithm for cSCC.

The role of recurrent disease also merits special attention. Multiple studies have shown that recurrent tumors, regardless of size or depth of invasion, behave more aggressively and are associated with poorer outcomes, including higher rates of nodal spread and recurrence. Recurrence should be considered high-risk, warranting closer surveillance or intervention [70,71,72].

### 4.5. Limitations

This review has several important limitations. Many studies had small sample sizes and substantial heterogeneity, particularly in inclusion criteria, clinical outcome measures, and reporting standards, which made pooled analysis difficult. We acknowledge that this heterogeneity extended beyond study design to include inconsistencies in risk definitions and nodal staging. Several studies used different or undefined criteria for “high-risk” disease, while others did not report tumor characteristics at all. These differences have likely contributed to the variability in outcomes and should be considered when interpreting pooled results. For example, pooled demographic and tumor characteristic results should be interpreted with caution because of the wide confidence intervals in some variables (e.g., prevalence of PNI in the observed group was 53.3%, 95% CI: 0.3–100.0%).

Risk stratification methods were also inconsistent with some studies not specifying which factors defined “high-risk” and others omitting classification altogether. In addition, occult metastasis rates may have been underestimated, as it was often unclear whether cases of tumor recurrence were included in the reported number of subclinical nodal metastases. Similarly, the determination of cN0 status was variably defined and inconsistently reported. In most studies, authors did not clearly describe the imaging modalities, physical examination protocols, or diagnostic thresholds used to ascertain nodal status. Moreover, radiologic and clinical assessments are known to vary across institutions and between reviewers, introducing additional heterogeneity.

Most of the studies included in our review were retrospective in design, and the relatively lower number of prospective studies limits the ability to make definitive conclusions. A sub-analysis limited to prospective studies was not feasible given the small number of available studies (2 in the observation group, 4 in the SLNB group, and 1 in the ED group) and sparse outcomes data. As such, prospective and retrospective data were combined for analysis to provide the most comprehensive review possible.

### 4.6. Future Directions

As published literature is primarily retrospective studies, future research should prioritize high-quality, prospective multicenter trials comparing observation, SLNB, ED, and other management strategies in risk-stratified cohorts. The evidence for elective neck irradiation remains sparse, precluding firm conclusions over its use. Further research is needed to clarify the role of elective neck irradiation in these populations.

## 5. Conclusions

This systematic review and meta-analysis found an overall occult nodal metastasis rate of 13.9% in clinically node-negative cutaneous squamous cell carcinoma of the head and neck. Sentinel lymph node biopsy was associated with lower recurrence and mortality compared with observation and elective dissection, supporting its role as a valuable prognostic and staging tool in this population. However, these findings should be interpreted as associations rather than evidence of a direct therapeutic effect. The apparent benefit of SLNB likely reflects earlier detection of subclinical disease and improved staging accuracy in appropriately selected patients. Underlying tumor biology, patient selection, and study-level confounding remain important factors that may influence these outcomes.

## Figures and Tables

**Table 1:** Characteristics of included studies.

Study Title	Author	Year	Country	Study Design	Management Strategy
Elective Neck Dissection Versus Observation in Patients With Head and Neck Cutaneous Squamous Cell Carcinoma	Amit	2021	USA	Retrospective cohort	Observation, ED
Elective Neck Dissection for Head and Neck Cutaneous Squamous Cell Carcinoma with Skull Base Invasion	Cannon	2017	USA	Retrospective cohort	Observation, ED
Lymphatic Mapping and Sentinel Lymphadenectomy for 106 Head and Neck Lesions: Contrasts Between Oral Cavity and Cutaneous Malignancy	Civantos	2006	USA	Prospective case series	SLNB
Sentinel lymph node biopsy with a gamma probe in patients with high-risk cutaneous squamous cell carcinoma	Demir	2011	Turkey	Prospective case series	^1^
Relevance of Intraparotid Metastases in Head and Neck Skin Squamous Cell Carcinoma	Dur	2020	Switzerland	Retrospective cohort	^1^
Sentinel Lymph Node Biopsy for Cutaneous Squamous Cell Carcinoma on the Head and Neck	Durham	2016	USA	Retrospective case series	SLNB
Clinical characteristics of malignant tumours originating in the external ear	Gallegos-Hernandez	2015	Mexico	Retrospective cohort	^1^
Prospective study of sentinel node biopsy for high-risk cutaneous squamous cell carcinoma of the head and neck	Gore	2016	Australia	Prospective case series	SLNB
Pattern of nodal metastasis of cutaneous squamous cell carcinoma involving the temporal bone	Hintze	2022	Ireland	Retrospective case series	^1^
The value of Elective Parotidectomy in Advanced Squamous Cell Carcinoma of the Skin of the Head	Hoch	2014	Germany	Retrospective case series	ED
Elective parotidectomy and neck dissection are not beneficial in cutaneous squamous cell carcinoma of the head	Horakova	2023	Czech Republic	Retrospective cohort	ED
The prognostic value of sentinel lymph nodes on distant metastasis–free survival in patients with high-risk squamous cell carcinoma	Jansen	2019	Germany	Ambispective cohort	^1^
The role of elective superficial parotidectomy in the treatment of temporal region squamous cell carcinoma	Kadakia	2015	USA	Retrospective cohort	ED
Indications for and extent of elective neck dissection for lymph node metastasis from external auditory canal carcinoma	Kiyokawa	2020	Japan	Retrospective cohort	^1^
The role of suprahyoid neck dissection in the treatment of the lower lip: 20 years experience at a Tertiary Center	Kuscu	2016	Turkey	Retrospective cohort	^1^
Availability of sentinel lymph node biopsy for cutaneous squamous cell carcinoma	Maruyama	2016	Japan	Retrospective cohort	^1^
Imaging and sentinel lymph node biopsy in high risk head and neck cutaneous squamous cell carcinoma: a Chinese cohort study	Ma	2025	China	Retrospective cohort	SLNB
Parotidectomy and neck dissection in locally advanced and relapsed cutaneous squamous cell carcinoma of the head and neck region	Melo	2022	Brazil	Retrospective cohort	ED
Sentinel Node Biopsy in 105 High-Risk Cutaneous SCCs of the Head and Neck: Results of a Multicenter Prospective Study	Mooney	2019	Australia	Prospective case series	SLNB
Lymph Node Metastases from Cutaneous Squamous Cell Carcinoma of the Head and Neck	Moore	2005	USA	Prospective cohort	^1^
Patterns of Regional Metastasis in Advanced Stage Cutaneous Squamous Cell Carcinoma of the Auricle	Peiffer	2011	USA	Retrospective case series	^1^
Sentinel Node Lymphoscintigraphy in High-risk Cutaneous Squamous Cell Carcinoma	Pollock	2017	USA	Prospective case series	^1^
Outcomes of Sentinel Lymph Node Biopsy for Primary Cutaneous Squamous Cell Carcinoma of the Head and Neck	Pride	2022	USA	Retrospective case series	SLNB
Intraoperative use of Mohs’ surgery for the resection of major cutaneous head and neck cancer under general anaesthetic: Initial experiences, efficiency and outcomes	Ridha	2015	UK	Prospective case series	Observation
Concordant Surgical Treatment: Non-melanocytic Skin Cancer of the Head and Neck	Ryu	2017	South Korea	Retrospective case series	^1^
Sentinel Lymph Node Biopsy for High-Risk Nonmelanoma Skin Cancers	Sahn	2007	USA	Retrospective case series	SLNB
Surgical Treatment of Lip Cancer: Our Experience With 106 Cases	Salgarelli	2009	Italy	Retrospective case series	Observation
Lymph Node Metastasis in Cutaneous Head and Neck Squamous Cell Carcinoma	Silberstein	2015	Israel	Retrospective case series	Observation
Sentinel lymph node biopsy in cN0 squamous cell carcinoma of the lip: A retrospective study	Sollamo	2015	Finland	Retrospective case series	SLNB
Sentinel node biopsy for high-risk cutaneous squamous cell carcinoma	Takahashi	2014	Japan	Retrospective case series	SLNB
Sentinel node radiolocalisation and predictive value in lip squamous cell carcinoma	Tartaglione	2003	Italy	Prospective case series	SLNB
Squamous Cell Carcinoma of the Lip in Australian Patients: Definitive Radiotherapy Is an Efficacious Option to Surgery in Select Patients	Thanh Pham	2015	Australia	Retrospective case series	Observation
Sentinel Lymph Node Biopsy in High-Risk Cutaneous Squamous Cell Carcinoma: Analysis of a Large Size Retrospective Series	Tremblay-Abel	2021	Canada	Retrospective case series	^1^
Sentinel Node Biopsy for High-Risk Nonmelanoma Cutaneous Malignancy	Wagner	2004	USA	Retrospective cohort	^1^
Efficacy of elective nodal irradiation in skin squamous cell carcinoma of the face, ears, and scalp	Wray	2015	USA	Retrospective case series	^2^
Sentinel Lymph Node Biopsy for High-Risk Cutaneous Squamous Cell Carcinoma of the Head and Neck	Wu	2020	USA	Retrospective case series	SLNB
Comparison between wait-and-see policy and elective neck dissection in clinically N0 cutaneous squamous cell carcinoma of head and neck	Xiao	2018	China	Prospective cohort	Observation, ED
The problem of nodal disease in squamous cell carcinoma of the temporal bone	Zanoletti	2010	Italy	Retrospective case series	^1^

^1^ Study did not have extractable clinical outcomes but reported occult rate for meta-analysis; ^2^ Study was included in systematic review, but no other similar studies were identified for meta-analysis.

**Table 2:** JBI critical appraisal of case series and cohort studies.

Study ID	1	2	3	4	5	6	7	8	9	10	11	Total	OLE		
Civantos	Y	Y	Y	Y	N	Y	Y	Y	N	Y	N/A	8	4	JBI Questions for case series 1. Were there clear criteria for inclusion in the case series? 2. Was the condition measured in a standard, reliable way for all participants included in the case series? 3. Were valid methods used for identification of the condition for all participants included in the case series? 4. Did the case series have consecutive inclusion of participants? 5. Did the case series have complete inclusion of participants? 6. Was there clear reporting of the demographics of the participants in the study? 7. Was there clear reporting of clinical information of the participants? 8. Were the outcomes or follow-up results of cases clearly reported? 9. Was there clear reporting of the presenting site(s)/clinic(s) demographic information? 10. Was statistical analysis appropriate?	
Demir	Y	Y	Y	Y	N	Y	Y	Y	N	Y	N/A	8	4		
Dur	Y	Y	Y	Y	Y	Y	Y	Y	N	Y	N/A	9	4		
Durham	Y	Y	Y	Y	Y	Y	Y	Y	N	Y	N/A	9	4		
Gallegos-Hernandez	Y	Y	Y	Y	Y	N	N	Y	N	N	N/A	6	4		
Gore	Y	Y	Y	Y	Y	Y	N	Y	N	Y	N/A	8	4		
Hintze	Y	Y	Y	Y	Y	N	Y	Y	N	Y	N/A	8	4		
Hoch	Y	Y	Y	Y	Y	Y	Y	Y	N	N	N/A	8	4		
Horakova	Y	Y	Y	Y	N	Y	Y	Y	N	N	N/A	7	4		
Mooney	Y	N	Y	Y	N	Y	Y	Y	N	Y	N/A	7	4		
Peiffer	Y	Y	Y	Y	Y	Y	Y	Y	N	Y	N/A	9	4		
Pollock	Y	Y	Y	Y	Y	Y	Y	Y	N	Y	N/A	9	4		
Pride	Y	Y	Y	Y	N	Y	Y	Y	N	N	N/A	7	4		
Ridha	Y	Y	Y	Y	Y	N	Y	Y	N	Y	N/A	8	4		
Ryu	Y	Y	Y	Y	Y	Y	Y	N	N	N	N/A	7	4		
Sahn	Y	Y	N	Y	N	Y	Y	Y	N	N	N/A	6	4		
Salgarelli	N	Y	Y	Y	Y	N	Y	Y	N	N	N/A	6	4		
Silberstein	Y	Y	N	Y	Y	Y	Y	Y	N	Y	N/A	8	4		
Sollamo	Y	Y	Y	Y	Y	Y	Y	Y	N	Y	N/A	9	4		
Takahashi	Y	Y	Y	Y	N	Y	Y	Y	N	Y	N/A	8	4		
Tartaglione	Y	Y	Y	Y	N	Y	N	Y	N	N	N/A	6	4		
Thanh Pham	Y	Y	Y	Y	Y	Y	Y	Y	N	Y	N/A	9	4		
Tremblay-Abel	Y	Y	Y	Y	Y	Y	Y	Y	N	Y	N/A	9	4		
Wagner	Y	Y	Y	Y	N	Y	Y	Y	N	Y	N/A	8	4		
Wray	Y	Y	Y	Y	N	Y	Y	Y	N	Y	N/A	8	4		
Wu	Y	Y	Y	Y	Y	Y	Y	Y	N	Y	N/A	9	4		
Zanoletti	Y	Y	Y	Y	Y	N	Y	Y	N	Y	N/A	8	4		
Amit	Y	Y	Y	Y	Y	Y	Y	Y	Y	Y	Y	11	3	JBI Questions for cohort studies 1. Were the two groups similar and recruited from the same population? 2. Were the exposures measured similarly to assign people to both exposed and unexposed groups? 3. Was the exposure measured in a valid and reliable way? 4. Were confounding factors identified? 5. Were strategies to deal with confounding factors stated? 6. Were the groups/participants free of the outcome at the start of the study (or moment of exposure)? 7. Were the outcomes measured in a valid and reliable way? 8. Was the follow up time reported and sufficient to be long enough for outcomes to occur? 9. Was follow up complete, and if not, were the reasons to loss to follow-up described and explored? 10. Were strategies to address incomplete follow up utilized? 11. Was appropriate statistical analysis used?	
Cannon	Y	Y	Y	Y	Y	Y	Y	Y	Y	Y	Y	11	3		
Jansen	Y	Y	Y	Y	Y	Y	Y	Y	Y	N	Y	10	3		
Kadakia	Y	Y	Y	Y	Y	Y	Y	Y	Y	Y	Y	11	3		
Kiyokawa	Y	Y	Y	Y	N	Y	Y	Y	Y	N	Y	9	3		
Kuscu	Y	Y	Y	N	N	Y	Y	Y	Y	Y	Y	9	3		
Maruyama	Y	Y	Y	N	N	Y	Y	Y	N	N	Y	7	3		
Ma	Y	Y	Y	Y	Y	Y	Y	Y	Y	N	Y	10	3		
Melo	Y	Y	Y	Y	Y	Y	Y	Y	Y	Y	Y	11	3		
Moore	Y	Y	Y	Y	Y	Y	Y	Y	N	N	Y	9	3		
Xiao	Y	Y	Y	Y	N	Y	Y	Y	N	N	Y	8	3		

Abbreviations: Y—Yes; N—No; N/A—Not Applicable; JBI—Joanna Briggs Institute critical appraisal tool; OLE—Oxford Level of Evidence. The Total represents the sum of the number of “Yes” responses, with the decision to include if the rate of “Yes” responses exceeds 50%.

**Table 3:** Meta-Proportions of patient demographics and tumor characteristics.

	Observation	95% CI	SLNB	95% CI	ED	95% CI
Sample, n	1799	N/A	494	N/A	411	N/A
Tumors, n	1952	N/A	499	N/A	411	N/A
Age, years (SD)	69.3 (0.9)	67.5–71.0	68.3 (1.3)	65.7–70.8	68.3 (2.1)	64.2–72.4
Sex, % male	75.5	57.9–89.5	80.9	77.2–84.3	78.0	67.1–87.2
Follow-up period, months (SE)	26.5 (1.2)	24.2–28.8	26.8 (2.2)	22.5–31.2	37.6 (7.8)	22.3–52.9
Tumor Location, %						
Ear and periauricular ^1^	1.6	0.6–10.5	7.6	2.7–14.8	9.2	1.3–23.3
Face ^2^	2.8	0.4–14.9	6.5	1.9–13.7	3.9	0.5–10.6
Forehead ^3^	3.2	0.002–12.1	0.7	0.2–1.9	1.3	0.1–4.0
Nose	1.9	0.06–8.6	3.9	1.5–7.4	4.0	0.5–10.6
Orbital ^4^	1.6	0.0006–6.4	0.9	0.3–2.2	2.4	0.04–8.3
Perioral ^5^	35.4 *	3.5–78.3	25.7	8.9–47.6	0.6	0.09–2.0
Scalp	1.8	0.05–8.3	8.7	2.5–18.1	1.3	0.1–4.0
Neck	0.9	0.05–4.3	3.4	2.0–5.4	0.4	0.02–1.6
Temple	1.0	0.1–5.4	1.1	0.4–2.5	13.3 *	2.8–62.9
Unspecified	27.9 *	4.9–94.6	30.1 *	5.7–63.2	41.7 *	0.6–94.0
Tumor Characteristics, %						
AJCC stage T1–T2	73.3	48.9–91.9	33.2 *	2.4–77.3	56.4 *	27.7–82.9
AJCC stage T3–T4	25.4	5.6–53.2	66.8	22.8–97.7	43.6 *	17.1–72.3
Primary Tumor	83.4	53.8–99.1	92.5	81.8–98.7	52.9 *	13.9–89.9
Recurrent Tumor	16.6	0.9–46.2	6.3	0.7–17.0	47.1 *	10.1–86.1
Perineural Invasion	53.3 *	0.3–100.0	33.0	23.4–43.2	48.7 *	12.8–85.4
Lymphovascular Invasion	–		8.9	5.6–13.2	16.3	11.3–22.3
Adjuvant Therapy, %						
Chemoradiation	0.2	0.0006–0.8	3.0	0.7–6.7	10.7	3.1–22.0
Radiation	28.7 *	2.9–66.9	21.6	5.5–44.5	43.8 *	11.1–79.9
Chemotherapy	0.7	0.02–3.2	3.0	0.7–6.7	5.4	0.05–18.8

* Results should be interpreted carefully due to low sample sizes and variability in pooled data; ^1^ Group includes tumor locations labeled as preauricular, postauricular, ear, pinna, lobe, or external auditory canal; ^2^ Group includes tumor locations labeled as face, midface, cheek, or malar; ^3^ Group includes tumor locations labeled as forehead or frontal; ^4^ Group includes tumor locations labeled as periocular, eyelid, eyebrow, or orbit; ^5^ and Group includes tumor locations labeled as perioral, lip, commissure, or chin.

**Table 4:** Meta-proportions of occult rates by management strategy.

Intervention	Subgroup	Sample, n	Occult Rate, %	I^2^	95% CI
All Interventions	Overall	1673	13.9	75.4	10.5–17.7
	High risk only	977	12.5	72.4	8.5–17.0
SLNB	All SLNB	707	8.8	30.0	6.8–11.1
	High risk	631	8.4	30.0	6.3–10.8
	Unspecified risk	76	12.6	31.4	6.2–22.0
Elective Dissection	All ED ^1^	966	17.3	82.1	11.6–23.7
	High risk ^1^	346	18.8	75.5	10.3–29.1
	Unspecified risk ^1^	620	16.3	84.1	9.2–25.0
	Parotidectomy only	166	20.2	28.9	14.4–27.0

^1^ Group contains elective neck dissection and/or parotidectomy.

**Table 5:** Meta-proportions of clinical outcomes.

Outcome, %	Observation	95% CI	SLNB	95% CI	ED ^1^	95% CI
Local Recurrence	8.2	4.2–14.2	11.2	8.2–14.8	21.8	11.9–34.9
Regional Recurrence	6.7	3.2–11.4	6.8	4.5–9.7	7.6	5.1–10.8
Distant Metastasis	6.7	1.1–34.5	4.5	2.5–7.3	9.5	2.9–19.4
Overall Recurrence	16.9	4.9–34.2	8.3	2.1–18.1	23.7	15.1–33.7
Disease-Specific Death	5.0	0.7–12.9	5.6	3.5–8.3	6.7	0.1–22.9
5-year DFS	69.0	45.7–88.0	–	–	–	–
Overall Mortality	29.9	27.1–32.8	6.1	4.0–8.9	31.4	3.4–71.1

^1^ Group contains elective neck dissection and/or parotidectomy.

**Table 6:** Meta-proportions of outcomes and comparison of means and proportions.

	Obs. (n = 1799)	SLNB (n = 494)	ED ^1^ (n = 411)	Obs. vs. SLNB			Obs. vs. ED ^1^			SLNB vs. ED ^1^		
Patient Characteristics				∆	95% CI	*p*	∆	95% CI	*p*	∆	95% CI	*p*
Mean age, yr. (SE)	69.3 (0.9)	68.3 (1.3)	68.3 (2.1)	1.0	0.9–1.1	<0.0001	0.9	0.8–1.1	<0.0001	0.04	−0.2–0.3	0.7178
Sex, % male	75.5	80.9	78.0	5.4	1.2–9.3	0.0132	0.03	−2.3–6.9	0.2969	2.9	−2.5–8.4	0.2922
Mean follow up period, mo. (SE)	26.5 (1.2)	26.8 (2.2)	37.6 (7.8)	0.3	0.2–0.5	0.0001	11.1	10.7–11.4	<0.0001	10.7	10.0–11.5	<0.0001
Tumor characteristics, %												
AJCC stage T1–T2	73.3	33.2	56.4	40.2	33.6–46.2	<0.0001	16.9	11.1–22.9	<0.0001	23.2	14.9–31.1	<0.0001
AJCC stage T3–T4	25.4	66.8	43.6	41.4	34.9–47.4	<0.0001	18.2	12.4–24.1	<0.0001	23.2	14.9–31.1	<0.0001
Primary Tumor	83.4	92.5	52.9	9.1	3.4–12.8	0.0039	30.4	24.9–35.9	<0.0001	39.5	32.0–45.7	<0.0001
Recurrent Tumor	16.6	6.3	47.1	10.3	7.1–13.0	<0.0001	32.4	26.9–37.9	<0.0001	42.7	36.8–48.3	<0.0001
Perineural Invasion	53.3	33.0	48.7	20.3	9.4–30.9	0.0002	4.6	−7.3–16.3	0.4534	15.7	7.6–23.7	0.0001
Lymphovascular Invasion	–	8.9	16.3	–	–	–	–	–	–	7.4	1.1–14.1	0.0202
Outcomes, %												
Overall Recurrence	16.9	8.3	23.7	8.6	4.6–11.8	0.0002	6.8	1.6–12.7	0.0084	15.4	9.3–21.7	<0.0001
Local Recurrence	8.2	11.2	21.8	3.0	−3.6–8.0	0.3373	13.6	2.8–26.9	0.0103	10.6	0.9–23.8	0.0287
Regional Recurrence	6.7	6.8	7.6	0.1	−2.3–3.2	0.9573	0.9	−1.7–4.3	0.5147	0.9	−2.9–4.7	0.6444
Distant Metastasis	6.7	4.5	9.5	2.2	−2.0–8.0	0.3306	2.9	−3.7–9.1	0.3662	5.1	0.5–10.7	0.0273
Disease-Specific Death	5.0	5.6	6.7	0.5	−1.8–3.5	0.6798	1.6	−1.0–5.1	0.2455	1.1	−2.4–4.9	0.5399
5-year DFS	69.0	–	–	–	–	–	–	–	–	–	–	–
Overall Mortality	29.9	6.1	31.4	23.8	19.9–27.3	<0.0001	1.5	−4.9–8.4	0.6570	25.3	19.0–32.0	<0.0001

^1^ Group contains elective neck dissection and/or parotidectomy; – denotes insufficient data available for pooled analysis.

## Data Availability

The data that support the findings of this study are available from the corresponding author upon reasonable request.

## Abbreviations

The following abbreviations are used in this manuscript:

AJCC	American Joint Committee on Cancer
BWH	Brigham and Women’s Hospital
CI	Confidence interval
cN0	Clinically node-negative
COP	Comparison of proportions
cSCC	Cutaneous squamous cell carcinoma
DOI	Depth of invasion
ED	Elective dissection
ENI	Elective nodal irradiation
IHC	Immunohistochemistry
JBI	Joanna Briggs Institute
HNcSCC	Head and neck cutaneous squamous cell carcinoma
NCCN	National Comprehensive Cancer Network
OLE	Oxford Level of Evidence
PNI	Perineural invasion
PRISMA	Preferred Reporting Items for Systematic Review and Meta-Analysis
SLNB	Sentinel lymph node biopsy

## Appendix A

**Table A1:** Search terms for management of cN0 HNcSCC.

Database	Terms	Results, n
PubMed	(“squamous cell carcinoma of head and neck” OR “cutaneous squamous cell carcinoma” OR “cSCC” OR “skin squamous cell carcinoma”) AND (“clinically node-negative” OR “node-negative” OR “cN0” OR “no nodal metastasis”) AND (“head and neck” OR “head” OR “neck” OR “face” OR “ear” OR “pinna” OR “nose” OR “lip” OR “scalp” OR “cheek” OR “forehead” OR “chin” OR “periorbital” OR “temple” OR “eyelid” OR “jaw” OR “mandible” OR “maxilla” OR “perinasal” OR “oral commissure” OR “external auditory canal” OR “preauricular” OR “postauricular” OR “otolaryngology”) AND ((“SLNB” OR “sentinel lymph node” OR “sentinel node”) OR (“neck dissection” OR “elective neck” OR “cervical lymphadenectomy” OR “END” OR “node dissection”) OR (“surveillance” OR “observation” OR “non-operative”) OR (“radiation” OR “irradiation” OR “radiotherapy”)) AND (“outcomes” OR “mortality” OR “survival” OR “recurrence” OR “metastasis” OR “morbidity” OR “disease-free”)	388
SCOPUS	PubMed search terms adapted with syntax modifications	967
CINAHL	PubMed search terms adapted with syntax modifications	101
Web of Science	PubMed search terms adapted with syntax modifications	27

**Table A2:** Search terms for occult metastasis of cN0 HNcSCC.

Database	Terms	Results, n
PubMed	(“cutaneous squamous cell carcinoma” OR “cSCC” OR “skin squamous cell carcinoma”) AND (“head and neck” OR “head” OR “neck” OR “face” OR “ear” OR “pinna” OR “nose” OR “lip” OR “scalp” OR “cheek” OR “forehead” OR “chin” OR “periorbital” OR “temple” OR “eyelid” OR “jaw” OR “mandible” OR “maxilla” OR “perinasal” OR “oral commissure” OR “external auditory canal” OR “preauricular” OR “postauricular” OR “otolaryngology”) AND (“occult” OR “micrometastasis” OR “subclinical” OR “undiagnosed nodal involvement” OR “nodal upstaging”)	47
SCOPUS	PubMed search terms adapted with syntax modifications	1326
CINAHL	PubMed search terms adapted with syntax modifications	186
Web of Science	PubMed search terms adapted with syntax modifications	60
